# Passive amylin immunotherapy improves brain function and reduces brain β‐amyloid pathology in APP/PS1 mice

**DOI:** 10.1002/alz.095662

**Published:** 2025-01-09

**Authors:** Deepak Kotiya, Florin Despa

**Affiliations:** ^1^ University of Kentucky, Lexington, KY USA

## Abstract

**Background:**

Amylin is a systemic hormone that is co‐secreted with insulin from pancreatic β‐cells. Amylin co‐aggregates with brain parenchymal and vascular β‐amyloid in persons with Alzheimer’s dementia. The present pilot study sought to assess the safety and side effects during and after the treatment period of passive amylin immunotherapy in the APP/PS1 mouse model of Alzheimer’s disease.

**Method:**

We have generated a polyclonal anti‐amylin antibody (P2) in rabbits using the N‐terminal of amylin peptide as an immunogen. In this pilot study at 5 months of age, male APP/PS1 mice were randomly assigned into rabbit Ig‐G vehicle‐control and P2 amylin antibody injected group (n = 4/group). Anti‐amylin antibody was injected at a dose of 10 mg/kg of body weight three times a week for six weeks. During the six weeks of amylin immunotherapy, we monitored the mice for visible side effects and immunocomplex disease‐related visible symptoms. Six weeks later, we assessed body weight, blood glucose level, and brain function with the novel object recognition test and performed comparative immunochemical Aβ analyses of hippocampal tissue by using MSD ELISA.

**Result:**

During the period of passive amylin immunotherapy, we did not observe visible symptoms of immunocomplex disease such as rash, urticaria, and malaise along with any sudden drop in body weight. Mice with anti‐amylin antibody injected showed an enhanced trend of recognition memory index (p = 0.1121) along with a lower blood glucose level (p = 0.2602) as compared to those that were injected with rabbit Ig‐G vehicle control. This was associated with decreasing trend of hippocampal levels of Aβ42 (p = 0.7872), Aβ40 (p = 0.4609), Aβ38 (p = 0.4473) in brain sup fraction, and Aβ42 levels (p = 0.6336) in brain pellet fraction measured by MSD ELISA.

**Conclusion:**

Passive amylin immunotherapy in APP/PS1 mice shows that used immunotherapy procedure might be safe for further investigation. Future experiments will be performed in APP/PS1 mouse model in which the mouse amylin gene is replaced by the human amylin gene in the pancreas and is conditionally downregulated by tamoxifen (TMX) injection, intraperitoneally.

